# Pharmacokinetics and Pharmacodynamics of an Oral Formulation of Apixaban in Horses After Oral and Intravenous Administration

**DOI:** 10.3389/fvets.2018.00304

**Published:** 2018-12-04

**Authors:** Priscila B. S. Serpa, Marjory B. Brooks, Thomas Divers, Sally Ness, Ingvild Birschmann, Mark G. Papich, Tracy Stokol

**Affiliations:** ^1^Department of Population Medicine and Diagnostic Science, College of Veterinary Medicine, Cornell University, Ithaca, NY, United States; ^2^Department of Clinical Sciences, College of Veterinary Medicine, Cornell University, Ithaca, NY, United States; ^3^Institut für Laboratoriums-und Transfusionsmedizin, Herz-und Diabeteszentrum Nordrhein-Westfalen, Universitätsklinik der Ruhr-Universität Bochum, Bad Oeynhausen, Germany; ^4^Department of Molecular Sciences, College of Veterinary Medicine, North Carolina State University, Raleigh, NC, United States

**Keywords:** factor Xa, direct oral anticoagulant, EHV-1, platelet activation, equine

## Abstract

Horses with inflammatory and infectious disorders are often treated with injectable heparin anticoagulants to prevent thrombotic complications. In humans, a new class of direct oral acting anticoagulants (DOAC) appear as effective as heparin, while eliminating the need for daily injections. Our study in horses evaluated apixaban, a newly approved DOAC for human thromboprophylaxis targeting activated factor X (Xa). Our goals were to: (1) Determine pharmacokinetics and pharmacodynamics of apixaban after oral (PO) and intravenous (IV) administration in horses; (2) Detect any inhibitory effects of apixaban on *ex vivo* Equid herpesvirus type 1 (EHV-1)-induced platelet activation, and (3) Compare an anti-Xa bioactivity assay with ultra-performance liquid chromatography-mass spectrometry (UPLC-MS) for measuring apixaban concentrations. In a blinded placebo-controlled cross-over study, five horses received a single dose (0.2 mg/kg) of apixaban or placebo PO or IV. Blood was collected before and at 3 (IV) or 15 (PO) min, 30 and 45 min, and 1, 2, 3, 4, 6, 8, and 24 h after dosing for measuring apixaban UPLC-MS concentrations and anti-Xa activity. Pharmacodynamic response was measured in a dilute prothrombin time (dPT) assay. Flow cytometric EHV-1-induced platelet P-selectin expression and clinical pathologic safety testing were performed at baseline, 2 and 24 h and baseline and 24 h, respectively. We found no detectable apixaban in plasma PO administration. After IV administration, plasma apixaban levels followed a two-compartment model, with concentrations peaking at 3 min and decreasing to undetectable levels by 8 h. The elimination half-life was 1.3 ± 0.2 h, with high protein binding (92–99%). The dPT showed no relationship to apixaban UPLC-MS concentration and apixaban did not inhibit EHV-1-induced platelet activation after IV dosing. Apixaban anti-Xa activity showed excellent correlation to UPLC-MS (*r*^2^ = 0.9997). Our results demonstrate that apixaban has no apparent clinical utility as an anticoagulant for horses due to poor oral availability.

## Introduction

Inherited and acquired diseases can cause abnormal hemostasis in horses, leading to either insufficient or excessive coagulation or fibrinolysis, resulting in hemorrhage or thrombosis (1). Hypercoagulable states place affected animals at risk of thrombosis with its clinical sequela of ischemic tissue injury, organ dysfunction and possible end-organ failure (2). Hypercoagulable states are usually caused by bacterial sepsis (3) or inflammatory gastrointestinal conditions (4) in horses, but thrombosis may also contribute to the pathogenesis of laminitis (5, 6) and acute renal failure (7). Equid herpesvirus type 1 (EHV-1) is a highly contagious and worldwide distributed virus that causes respiratory disease, neurologic disease and abortion (8). The virus infects endothelial cells, resulting in vasculitis and thrombi, with associated ischemic and inflammatory lesions in the central nervous system and placenta (9, 10). We and others have shown that EHV-1 is associated with a hypercoagulable state in horses, as seen by increased D-dimer concentrations in experimentally infected horses (11) and the ability of the virus to upregulate tissue factor on monocytes (12) and generate thrombin, leading to platelet activation, *ex vivo* (12).

Due to the serious clinical consequences of thrombosis, horses that are considered at risk of or suffering from thrombosis are often treated with antithrombotic drugs. Current antithrombotic therapy in horses is limited to oral warfarin (13), the injectable heparins (unfractionated heparin and low-molecular-weight heparin) (14–16), and antiplatelet drugs, such as aspirin (17) and clopidogrel (18, 19). Although anticoagulants, such as warfarin and heparin are effective, they can result in excessive bleeding and heparin can cause red blood cell agglutination and anemia and localized swellings at injection sites (15, 20, 21). They also require coagulation monitoring due to variable responses between individuals (14, 15).

Direct oral anticoagulants (DOAC), which target specific coagulation factors, have recently gained Federal Department of Agriculture approval in the USA. Apixaban, edoxaban, and rivaroxaban act as specific inhibitors of activated factor X (Xa). These DOACs demonstrate similar efficacy to low-molecular-weight heparin and are associated with less severe adverse bleeding events compared to warfarin, the most commonly used anticoagulant in people (22, 23). Their anticoagulant action is also reversible (24). Apixaban has been tested for anticoagulant activity in cats, dogs, mice, rabbits, and rats (25–28). In these species, the drug is bioavailable and demonstrates anti-Factor Xa (anti-Xa) activity. Rivaroxaban has also been experimentally tested in cats (29) and dogs (30), however only one published small case series describes its clinical use in dogs (31).

An unidentified direct oral anti-Xa inhibitor was administered to nine horses at a dose of 0.125 mg/kg PO at 24 h intervals for 4 days, however prophylactic anti-Xa activity was detected in only one animal after the first dose (32). To our knowledge, apixaban has not yet been tested in horses. Since it is an orally active anticoagulant, its use in horses would be desirable to avoid adverse effects related to parenteral administration of the heparin-based drugs, such as injection site swelling and hematoma. Therefore, the objectives of this study were to: (1) Measure the pharmacokinetics of an oral tablet formulation of apixaban after oral and intravenous (IV) administration to healthy horses; (2) Determine if apixaban was effective at inhibiting EHV-1-induced platelet activation *ex vivo*; and (3) Compare apixaban concentrations measured with an apixaban calibrated anti-Xa activity assay to that of ultra-performance liquid chromatography-mass spectrometry (UPLC-MS).

## Material and Methods

### Animals

Five clinically healthy horses were used in this trial. They were group-housed at the Equine Park at Cornell University. The horses were transported the day prior to drug administration to the Cornell University Hospital for Animals facilities and placed in individual stalls. They consisted of one gelding and four mares, ranging from 15 to 19 years of age, 511–626 kg bodyweight, and were from four different breeds (two Oldenburgs, and one each of Holsteiner, Quarter Horse, and Warmblood).

### Drug Preparation

For the preparation of the oral formulation, apixaban tablets (Eliquis®, Bristol-Myers Squibb, Pfizer, New York, NY, USA) were crushed with a mortar and pestle and the powder was mixed with 70 g of molasses (Herdlife™, New Roads, LA, USA). The paste was transferred to a 60 mL syringe (Becton-Dickinson) and stored at room temperature until administration. For IV formulation, crushed tablets were dissolved in a vehicle consisting of 10% v/v N,N-dimethylacetamide (EMD Millipore, Billerica, MA, USA), 17% v/v propylene glycol (Sigma-Aldrich), 17% v/v dimethyl sulfoxide (DMSO, Sigma-Aldrich), and 56% v/v deionized water (MilliQ® water, Q-Pod®, EMD Millipore). After vigorous vortexing, the formulation was sterile filtered through a 0.22 μM cellulose acetate filter (Corning Inc., Corning, NY, USA) then drawn into 60 mL syringes. The IV formulation was prepared 24 h before administration and stored at room temperature. We aimed for a final concentration of 2.5 mg/mL apixaban in the IV formulation. Since each tablet of Eliquis® weighed 210 mg, equal weights of lactose (Blackburn Distributions, Nelson, Lancashire, UK) and microcrystalline cellulose 102 (Blackburn Distributions) were crushed to create a placebo, which was similarly mixed with molasses or vehicle diluents for oral and IV administration, respectively, and stored as for the apixaban formulations. To ensure that the vehicle was not toxic, safety testing was performed on a trial IV dose given to a different horse. The horse had no adverse clinical reactions and there were no changes in clinical pathologic test results 24 h after IV administration of the bolus of dissolved apixaban.

### Study Design and Blood Collection

**Figure 1:**
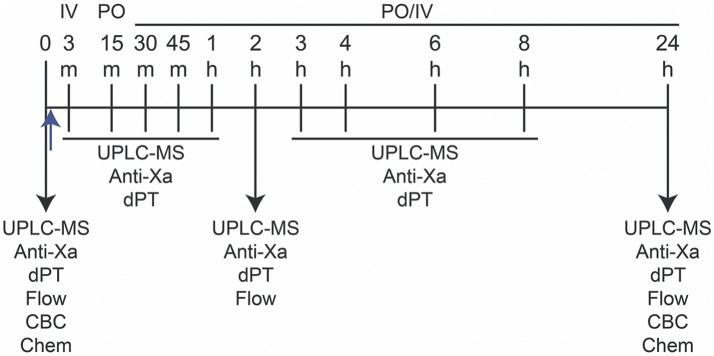
Study design for measurement of apixaban pharmacokinetics and pharmacodynamics in five horses. Apixaban (0.15–0.2 mg/kg) or placebo were administered orally (per os, PO) or intravenously (IV) just after baseline (0) blood samples were taken (upward blue arrow). The PO or IV routes were randomized for each horse in the following order: IV, PO, PO, IV, with each administration being followed by a 14-day washout. Blood samples were collected into 3.8% citrate anticoagulant at 0, 3 (IV only), 15 (PO only), 30, and 45 minutes (min), and 1, 2, 3, 4, 6, 8, and 24 hours (h) after drug or placebo administration for ultra-performance liquid chromatography-mass spectrometry (UPLC-MS) and anti-activated factor X (Anti-Xa) activity and dilute prothrombin time (dPT) assays on platelet-poor plasma. Flow cytometry (Flow) for quantification of P-selectin expression in agonist-activated platelets was performed at 0, 2, and 24 h in citrate-anticoagulated platelet-rich plasma. Blood was collected into EDTA- and non-anticoagulant vacutainer tubes at 0 and 24 h for a complete blood count (CBC) and select biochemical (Chem) tests, respectively.

In this double-blinded placebo-controlled cross-over study, apixaban or placebo was given either per os (PO) or by IV injection to horses at 0.2 mg/kg, followed by a 14-day washout between treatments. This dose was chosen based on the pharmacokinetic parameters after oral and IV administration to cats, which resulted in a C_max_ of 74 and 460 ng/mL, respectively (25). The first and last treatments for each horse were IV administration of drug or placebo (Figure 1). Personnel, who were not involved in preparing and administering the drugs, data acquisition, or data analysis, randomized the oral or IV treatments for each horse between placebo and apixaban. Investigators were blinded to each treatment until the conclusion of the study.

Blood samples were collected before drug administration (time 0) and at 3 (IV) or 15 (PO) min, then at 30, 45 min, 1, 2, 3, 4, 6, 8, and 24 h after drug administration into 6-mL syringes (Monoject Syringe, Covidien Ltd, Mansfield, MA, USA) prefilled with 3.8% citrate anticoagulant (Sigma-Aldrich, St Louis, MO, USA) maintaining a ratio of 9:1 blood:citrate. The citrate-anticoagulated blood was used to prepare platelet-poor plasma (PPP) for measurement of UPLC-MS concentrations, anti-Xa activities and dilute prothrombin times (dPT) and platelet-rich plasma (PRP) for flow cytometric analysis of *ex vivo* platelet activation in response to exposure to EHV-1. Blood was also collected into EDTA- and non-anticoagulant samples for clinical pathologic testing before and 24 h after each treatment (Figure 1). With the exception of 24 h samples, blood was collected at each time point through an indwelling jugular catheter (14 G × 15 cm, Milacath®, MILA International, Florence, KY, USA) after removing 20–30 mL of blood. Patency of the catheters was maintained by flushes with sterile saline solution (0.9% sodium chloride injection USP, Baxter Healthcare Corporation, Deerfield, IL, USA) after each blood draw. The catheter was removed after collection of the 8 h sample. For IV administration, a second catheter was placed in the opposite jugular vein for bolus drug delivery and removed immediately after drug administration and flushing of the catheter with sterile saline. For the 24 h sample, blood was collected from the jugular vein (the vein contralateral to that used for the indwelling catheter) into a 3.8% citrate anticoagulant prefilled 6-mL syringe via an 18 G needle (Monoject Veterinary Needle, Covidien Ltd, Mansfield, MA, USA) and into EDTA- and non-anticoagulant vacutainers (Becton-Dickinson, Franklin Lakes, NJ, USA) via a vacutainer needle (21 G, Becton-Dickinson Eclipse™ Vacutainer® with pre-attached holder).

### Protein Binding of Apixaban

To measure the extent to which apixaban was bound to protein, previously frozen PPP from each horse from the first baseline sample was thawed in a 37°C waterbath and centrifuged at 16,000 × *g* for 5 min at room temperature. The supernatant was filtered with a 0.22 μM syringe filter (Corning Inc.). The PPP was then spiked with apixaban at 200 and 1,000 nM (equivalent to 92 and 460 ng/mL) using the same IV preparation that was administered to the horses. Half of the sample was subsequently frozen at −20°C and the rest was ultracentrifuged at 16,000 × *g* for 30 min in a molecular exclusion device (Amicon Ultra 30k filter, Millipore), to remove the major plasma proteins. The ultrafiltrate was then frozen at −20°C. Both the spiked plasma and the ultrafiltrate of the spiked plasma were analyzed for apixaban using UPLC-MS. The percentage of protein binding was calculated by the difference between the apixaban concentration in the spiked plasma and the ultrafiltrate divided by the concentration in the spiked plasma.

### Measurement of Apixaban Concentrations by Ultra Performance Liquid Chromatography-Tandem Mass Spectrometry

This was performed as previously described (33). Analysis by UPLC-MS/MS was performed on a 2D UPLC system (Waters Acquity UPLC H-class with 2D Technology System, Waters GmbH, Eschborn, Germany) directly coupled to a Xevo TQ-S tandem mass spectrometer (Waters GmbH, Eschborn, Germany) which was operated in electrospray positive ionization mode. The system control and data acquisition were performed using MassLynx NT 4.1 software with automated data processing by the MassLynx QuanLynx program provided with the instrument. The lower limit of detection for all DOACs of the UPLC-MS/MS method was < 0.2 ng/mL. Since DOAC values < 7 ng/ml were not clinically relevant, all time points below this value were considered zero.

### Measurement of the Anticoagulant Activity of Apixaban

The anti-Xa activity of apixaban was measured in citrate-anticoagulated PPP by the Comparative Coagulation Laboratory in the Animal Health Diagnostic Center at Cornell University. The PPP was harvested from the supernatant of PRP after high-speed centrifugation (13,000 × *g*, Accuspin Micro, Thermo Scientific, Rockford, IL, USA) for 5 min. The PPP was frozen at −20°C and assays were performed in batches. The PPP was thawed at 37°C in a water bath before analysis with an automated coagulation analyzer (STA® Compact, Diagnostica Stago, Parsipanny, NJ, USA). The assay is configured with a bovine activated Factor X reagent added in excess to the test plasma and a chromogenic substrate of Factor Xa (STA®-Liquid anti-Xa, Diagnostica Stago). In this assay, residual, uninhibited Factor Xa cleaves the chromogenic substrate such that the inverse of the color change in the reaction mixture is proportional to the drug concentration in the test plasma. Results are expressed as ng/mL anti-Xa activity, based on the calibration standard containing known apixaban concentrations in human plasma (STA®-Apixaban Calibrator, Diagnostica Stago). Assay controls, consisting of apixaban spiked-human plasma (STA®-Apixaban Controls) were measured before each batch of test samples.

### Dilute Prothrombin Time Assay for Pharmacodynamic Analysis

In an attempt to determine pharmacodynamic profiles of apixaban after IV administration, a dPT assay was performed on PPP samples at all time points after IV injection of apixaban or placebo. The assay was configured with a rabbit brain thromboplastin reagent (Thromboplastin LI, Helena Diagnostics, Beaumont, TX) prediluted 1:2 in an imidazole buffer containing 0.025 M calcium chloride. The reagent was diluted to enhance its sensitivity to detect the presence of apixaban's anticoagulant effect (34, 35). Test results were reported as clotting time in seconds.

### Pharmacokinetic and Pharmacodynamic Analysis

Pharmacokinetic analysis was performed using a two-compartment open model with weighting of 1/y^2^ (Phoenix®, WinNonlin® software, Certara Inc., St. Louis, Missouri). Compartmental analysis of the data after administration of apixaban was calculated according the following formula:

$$\begin{array}{l} \text{C}=\text{A}{\text{e}}^{-\alpha \text{t}}+\text{B}{\text{e}}^{-\beta \text{t}} \end{array}$$

where C is the apixaban concentration, A is the distribution phase y-axis intercept, e is the base of the natural logarithm, t is time after administration, α is the distribution rate constant, B is the elimination phase y-axis intercept, and β is the elimination phase rate constant (terminal phase). Secondary parameters calculated include distribution (α) and elimination (β) half-lives (T½), microdistribution rate constants, area-under-the-curve (AUC), apparent volume of distribution at steady-state (V_SS_), systemic clearance (CL), and mean residence time (MRT).

The pharmacodynamic analysis was performed by plotting the drug concentration (apixaban measured by UPLC-MS) on the x-axis and response (dPT) on the y-axis to determine if a relationship existed.

### Flow Cytometric Detection of P-Selectin Expression

We used flow cytometric measurement of agonist-induced P-selectin expression as an *ex vivo* test for the inhibitory activity of apixaban on thrombin generation. This was only done on samples collected before and 2 and 24 h after drug administration (Figure 1). We chose the 2 h time point for flow cytometric analysis, because this was the time of peak concentration achieved after oral administration and concentrations were still high at this time, albeit not at peak, after IV administration in cats (25). Flow cytometric analysis was done as we have previously described (36). In brief, the citrate-anticoagulated blood was processed within 30 min of collection. Red blood cells were allowed to settle by gravity sedimentation at room temperature for 20 min. Then PRP was prepared by centrifugation of the resulting leukocyte-platelet-rich plasma at 250 × *g* at 21°C for 10 min. Thrombin at 0.15 U/mL (Sigma-Aldrich), bovine factor Xa at 0.1 and 1 μg/mL (Haematologic Technologies, Essex Junction, VT, USA) and two strains of EHV-1 were used as platelet agonists, and phosphate-buffered saline (PBS, Mediatec, Manassas, VA, USA) was used as a negative control. The strains of EHV-1 were Ab4 (propagated in rabbit kidney 13 cells) and RacL11 (propagated in equine kidney cells) and they were used at low and high concentrations equivalent to 1 × 10^6^ plaque forming units (PFU)/mL and 5 × 10^6^ PFU/mL, respectively. Both strains were isolated from cell lysates on a sucrose cushion via ultracentrifugation as we have previously described (12). One harvested isolate of each strain was used for the entire study.

Platelet-rich plasma was diluted at a 1:5 ratio in binding buffer (10 mM HEPES, 140 mM NaCl, 2.5 mM calcium chloride, 1 mM glycine–proline–arginine–proline, pH 7.4, all chemicals from Sigma-Aldrich), then was incubated for 10 min with the various agonists. The platelets were then labeled with an Alexa647-conjugated antibody against P-selectin (final concentration of 66 ng/mL, clone Psel.KO.2.7, Novus Biologicals LLC, Littleton, CO, USA) for 10 min at room temperature in the dark. The reaction was quenched with binding buffer and the samples were immediately analyzed with a flow cytometer (BD FACSCalibur, BD Biosciences, San Jose, CA, USA). For analysis, platelets were gated on their forward and side scatter characteristics and the percentage of P-selectin-positive platelets determined from histogram plots.

### Clinical Pathologic Testing

This was performed in the Clinical Pathology laboratory in the Animal Health Diagnostic Center at Cornell University and was done to determine if there were any apixaban-related effects on hematologic or select biochemical test results after PO or IV administration. Automated hemogram results were obtained from the EDTA-anticoagulated blood with a hematology analyzer (ADVIA 2120, Siemens Healthcare Diagnostics Inc., Tarrytown, NJ, USA). To verify the automated platelet counts, modified Wright's-stained blood smears were evaluated for platelet clumps by trained medical technologists. Biochemical analysis for renal analytes (serum urea nitrogen and creatinine concentrations), liver enzymes (sorbitol dehydrogenase, glutamate dehydrogenase, aspartate aminotransferase and gamma glutamyl transferase activities), protein-related analytes (total protein, albumin, and globulin concentrations), and the acute phase protein, serum amyloid A, were performed with an automated chemistry analyzer (Hitachi P modular, Roche Diagnostics, Indianapolis, IN, USA) using manufacturer's reagents, with the exception of sorbitol dehydrogenase (Sigma-Aldrich), glutamate dehydrogenase (Randox Laboratories Ltd, Antrim, UK), and serum amyloid A (LZ test Eiken-SAA, Mast House, Merseyside, UK).

### Statistical Analysis

Data was tested for normality using a Shapiro-Wilk normality test. Comparison of the mean or median of 2 groups was conducted with a paired *t*-test or Wilcoxon matched-pairs signed rank test, respectively. Multiple means or medians were compared with one-way ANOVA corrected for multiple comparisons with Tukey's *post-hoc* method or a Friedman test with Dunn's *post-hoc* method, respectively. Pearson correlation analysis was used to assess the association between apixaban plasma concentrations determined by UPLC-MS and the anti-Xa activity assay or the dPT. Alpha was set at 0.05. Statistical software used was Prism 7.03 for Windows (GraphPad Software Inc, La Jolla, CA, USA).

## Results

### Pharmacokinetic and Pharmacodynamic Analysis

**Figure 2:**
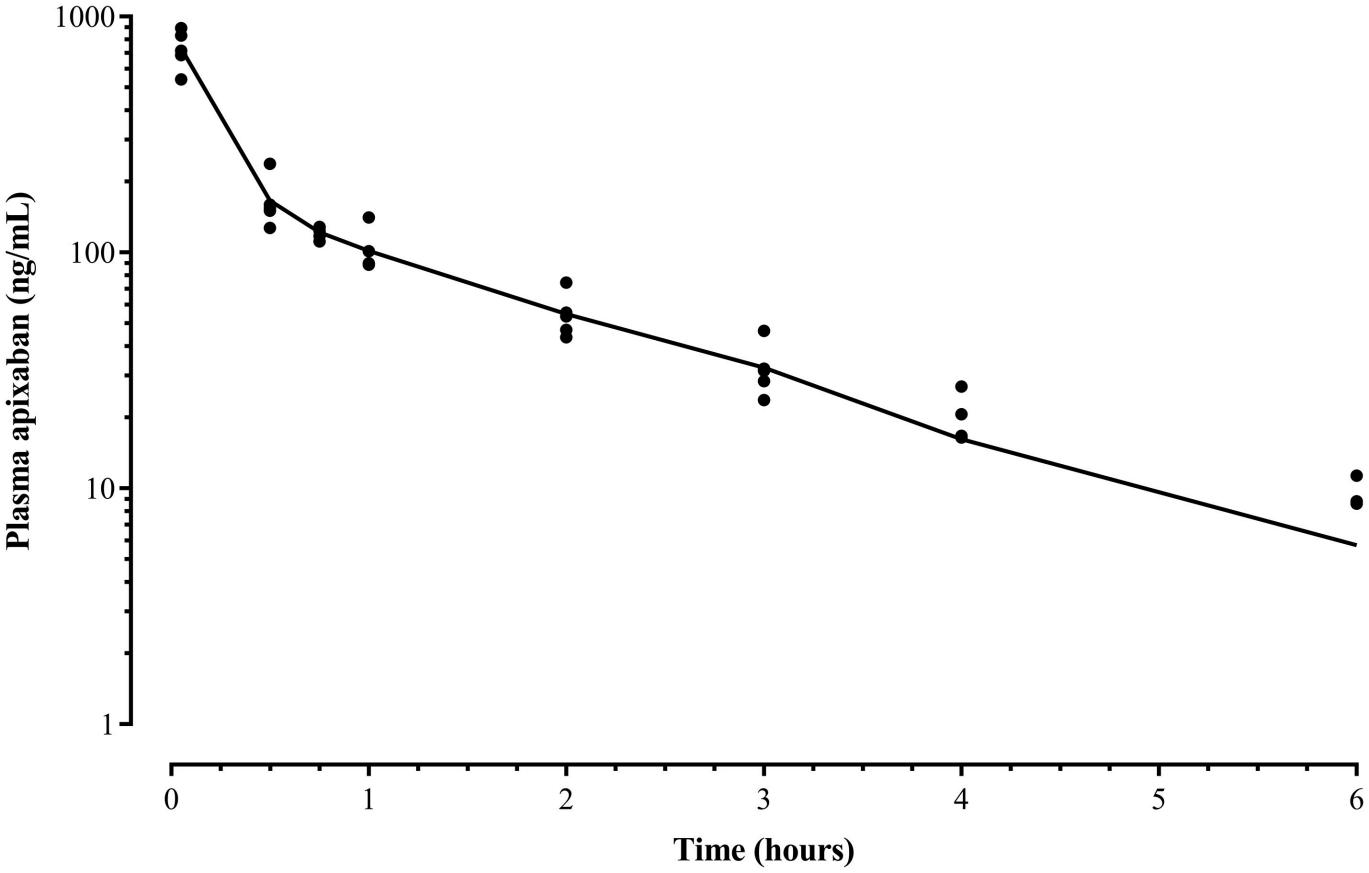
Mean apixaban plasma concentration (line) and individual data (•) after a bolus intravenous administration of 0.15 mg/kg to five horses before drug administration (time 0) and at 3, 30, 45 min, and 1, 2, 3, 4, 6, 8, and 24 h after drug administration measured by ultra performance liquid performance-mass spectrometry. The data represents a two-compartment pharmacokinetic curve, with a rapid initial phase of decline, followed by a slower and longer phase of elimination. Mean concentrations in samples collected after 6 h were below the detection limit of the assay.

Liquid chromatography data revealed that apixaban was not detected in plasma after oral administration. The mean apixaban concentration of the 5 IV formulations was 1.8 mg/mL (range, 0.84–2.6 mg/mL) reflecting a 28% loss of drug compared to the intended concentration of 2.5 mg/mL. Therefore, the average administered dose given to the 5 horses was 0.15 mg/kg instead of 0.2 mg/kg. A two-compartment model was found to be the best fit after IV administration for all five animals (Figure 2). The mean ± standard deviation (SD) plasma concentration of apixaban peaked at 733 ± 136 ng/mL at 3 min and decreased to undetectable levels by 8 h in all horses. The drug had a short elimination half-life of 1.3 ± 0.2 h and a low volume of distribution (VD_SS_ 93.3 ± 75.6 mL/kg) (Table 1). The mean percentage of protein binding for apixaban was 92.4 ± 0.5 and 98.5 ± 0% for 92 and 460 ng/mL, respectively.

**Table 1:** Mean, geometric mean, and standard deviation for compartmental analysis of pharmacokinetic parameters after single dose intravenous administration of 0.15 mg/kg apixaban to five horses.

**Parameter**	**Unit**	**Mean**	**Geometric mean**	**Standard deviation**	**Coefficient of variation (%)**
AUC	h·ng/mL	1,409.05	1,362.66	396.18	29.07
CL	mL/h/kg	151.99	146.77	45.55	31.03
A	ng/mL	14,340.87	12,003.59	8,461.26	70.49
α	h^−1^	12.21	11.89	3.11	26.20
t½α	h	0.06	0.06	0.02	27.82
B	ng/mL	171.11	170.28	19.14	11.24
β	h^−1^	0.55	0.54	0.09	16.40
t½β	h	1.28	1.27	0.18	14.47
K_10_	h^−1^	9.56	8.97	3.54	39.42
K_12_	h^−1^	2.48	2.43	0.52	21.26
K_21_	h^−1^	0.73	0.72	0.09	12.06
MRT	h	0.55	0.49	0.29	58.62
V_C_	mL/kg	20.11	16.36	15.37	93.93
V_SS_	mL/kg	93.27	71.85	75.58	105.20

*AUC, area under the curve; CL, systemic clearance; A, y-axis intercept of the distribution phase; α, rate constant of the distribution phase; t½α, half-life of the distribution phase; B, y-axis intercept of the elimination phase; β, rate constant of the elimination phase; t½β, half-life of the elimination phase; K_10_, elimination rate constant from the central compartment; K_12_, transfer rate constant between the central compartment and the peripheral compartment; K_21_, transfer rate constant between the peripheral compartment and the central compartment; MRT, mean residence time; V_C_, apparent volume of distribution for central compartment; V_SS_, volume of distribution at steady-state*.

**Figure 3:**
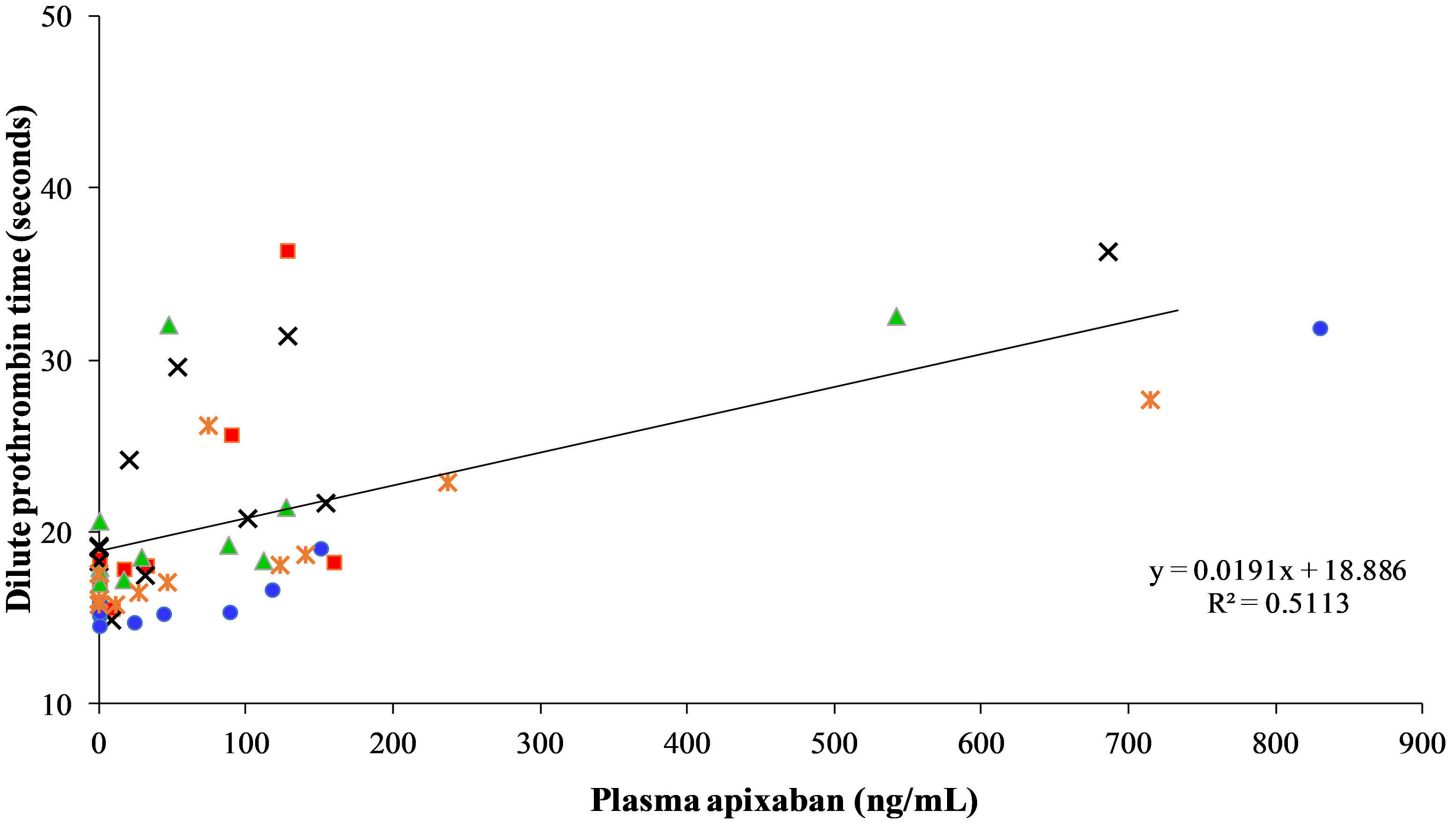
Correlation between dilute prothrombin time measured in seconds and apixaban concentration measured by ultra performance liquid performance-mass spectrometry measured in ng/mL at 0, 3, 30, and 45 min, and 1, 2, 3, 4, 6, 8, and 24 h after intravenous administration of apixaban at 0.15 mg/kg to five horses. Each horse has the same unique color and symbol at all time points for both treatments (horse 1: blue, •; horse 2: red, ■; horse 3: green, ▴; horse 4: orange, 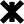; horse 5: black, ×).

There was no discernible relationship between the dPT and plasma apixaban concentrations measured by the UPLC-MS (Figure 3), precluding the use of this coagulation assay as the response for measuring pharmacodynamic parameters of apixaban. Individual horses showed substantial variability in their dPT over time, which contributed to the lack of an observed relationship.

### Inhibition of Platelet Activation

**Figure 4:**
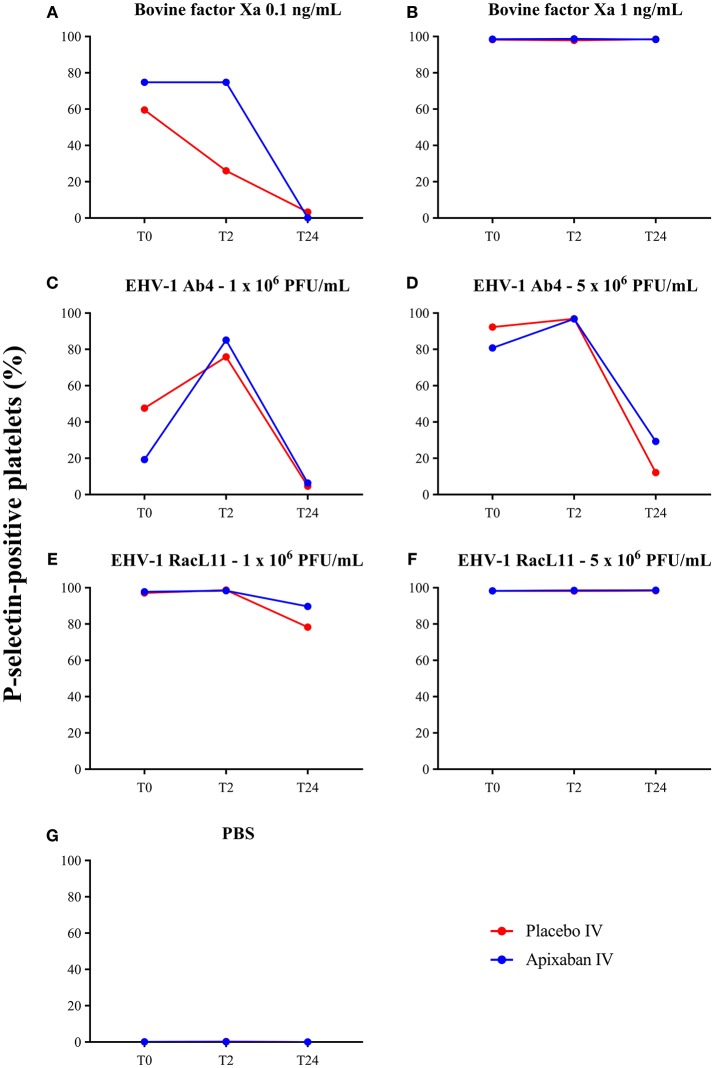
Changes in the median percentage of platelets expressing P-selectin before treatment (T0), and 2 (T2), and 24 (T24) h after oral administration of placebo (red) or apixaban (blue) at 0.2 mg/kg to five horses. Platelets were exposed to bovine factor Xa at low (0.1 ng/mL, **A**) and high (1 ng/mL, **B**) concentrations, low (1 × 10^6^ PFU/mL), and high (5 × 10^6^ PFU/mL) concentrations of EHV-1 strains, Ab4 (**C**, low concentration; **D**, high concentration) and RacL11 (**E**, low concentration; **F**, high concentration), or PBS as a negative control **(G)**.

**Figure 5:**
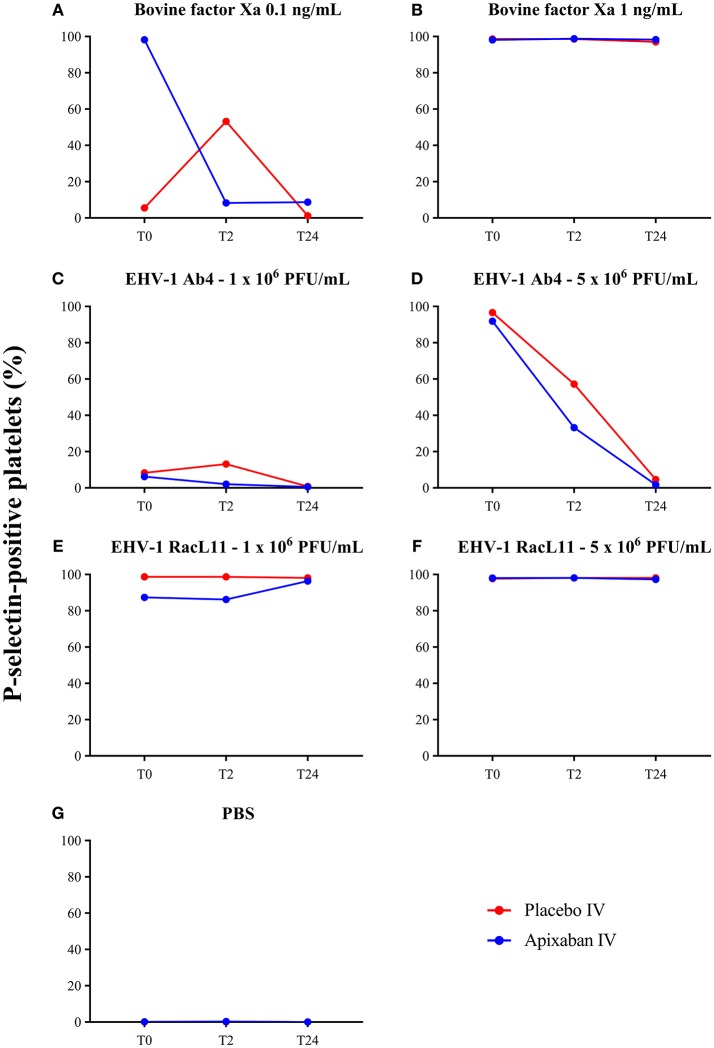
Changes in the median percentage of platelets expressing P-selectin before treatment (T0), and 2 (T2), and 24 (T24) h after intravenous administration of placebo (red) or apixaban (blue) at 0.15 mg/kg to five horses. Platelets were exposed to bovine factor Xa at low (0.1 ng/mL, **A**) and high (1 ng/mL, **B**) concentrations, low (1 × 10^6^ PFU/mL) and high (5 × 10^6^ PFU/mL) concentrations of EHV-1 strains, Ab4 (**C**, low concentration; **D**, high concentration) and RacL11 (**E**, low concentration; **F**, high concentration), or PBS as a negative control **(G)**.

**Figure 6:**
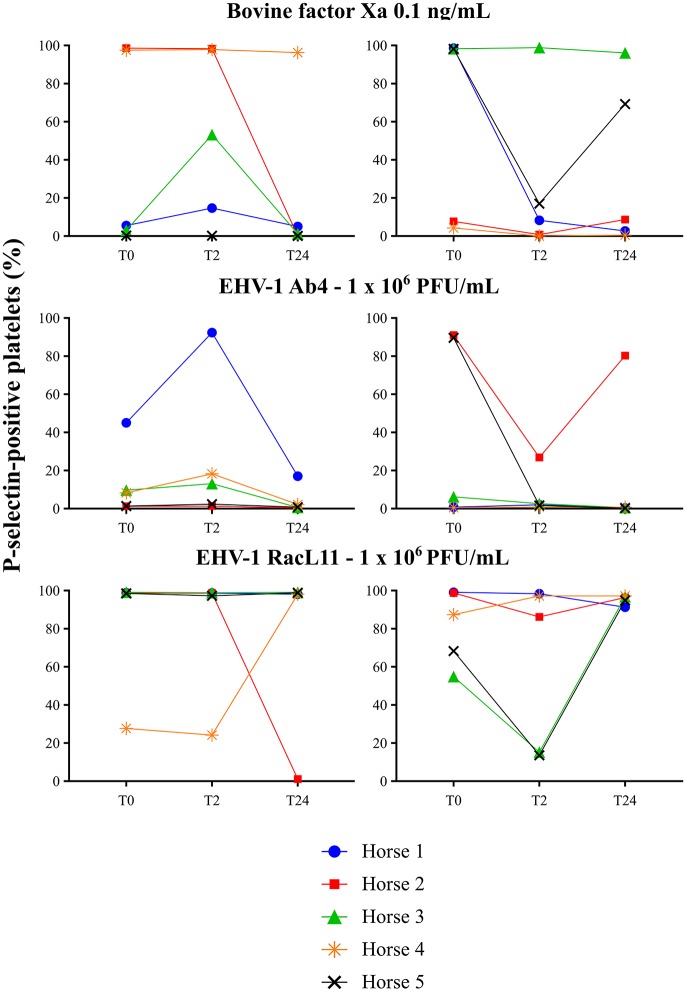
Individual changes in the 5 horses in the percentage of platelets expressing P-selectin in platelet-rich plasma exposed to low concentrations of bovine factor Xa (0.1 ng/mL) and EHV-1 strains Ab4 and RacL11 (1 × 10^6^/mL) before treatment (T0), and 2 (T2), and 24 (T24) h after intravenous administration of placebo (left panels) or 0.15 mg/kg apixaban (right panels). Each horse has the same unique color and symbol at all time points for both treatments (horse 1: blue, •; horse 2: red, ■; horse 3: green, ▴; horse 4: orange, 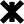 horse 5: black, ×).

There was no decrease in the median percentage of platelets expressing P-selectin in PRP when activation was induced by a high concentration of bovine factor Xa or low or high concentrations of the RacL11 EHV-1 strain at 2 h after PO or IV administration of apixaban (Tables 2, 3; Figures 4, 5). A decrease in the median percentage of platelets expressing P-selectin was seen in PRP exposed to low concentrations of bovine factor Xa and both concentrations of the Ab4 EHV-1 strain at 2 h after IV administration of apixaban. However, similar changes were seen in PRP stimulated with high concentrations of Ab4 at 2 h in animals given the IV placebo (Figure 5). In addition, platelets were either not reactive or minimally reactive to the low concentration of bovine factor Xa or both concentrations of Ab4 at 24 h, regardless of whether placebo or apixaban were administered PO or IV (Tables 2, 3; Figures 4, 5). These findings argue against any drug effect on platelet activation at the 2 h time point. Since median concentrations may mask individual results, we also examined changes in the individual horses with the low concentrations of each agonist after IV administration only. This data showed that platelet reactivity was reduced at 2 h in the PRP of four apixaban-dosed horses with the three agonists at low dose, with one horse (horses 5) showing decreases with all three agonists. In contrast, no decreases in platelet reactivity were seen in placebo-treated horses. However, in two out of the four horses, the changes in P-selectin expression were mild or still present at 24 h, making it difficult to attribute the decreases at 2 h to a drug effect (Figure 6). At the 2 h time point, the apixaban concentration ranged from 44 to 74 ng/mL in individual horses after IV administration. When we spiked equine PRP with similar concentrations of apixaban *ex vivo*, we did not see any inhibition of platelet activation (data not shown), further support for a lack of drug-induced inhibition of platelet reactivity at the 2 h time point.

**Table 2:** Median (range) percentage of platelets expressing P-selectin in platelet-rich plasma (PRP) from five horses before (T0) and 2 (T2) and 24 (T24) h after oral (PO) administration of placebo or 0.2 mg/kg apixaban.

**Agonist**	**P-selectin-positive platelets (%)**					
	**Placebo PO**			**Apixaban PO**		
	**T0**	**T2**	**T24**	**T0**	**T2**	**T24**
PBS	0 (0–0)	0 (0–1)	0 (0–0)	0 (0–0)	0 (0–1)	0 (0–0)
Factor Xa (0.1 ng/mL)	60 (0–99)	26 (0–99)	3 (0–11)	75 (0–99)	75 (0–99)	0 (0–96)
Factor Xa (1 ng/mL)	98 (98–99)	98 (96–99)	99 (81–99)	99 (97–99)	99 (98–99)	98 (96–99)
Ab4 (1 × 10^6^ PFU/mL)	48 (0–97)	76 (1–98)	5 (1–44)	19 (0–87)	85 (0–98)	6 (1–16)
Ab4 (5 × 10^6^ PFU/mL)	92 (1–98)	97 (2–98)	12 (3–96)	81 (1–97)	97 (1–97)	29 (1–89)
RacL11 (1 × 10^6^ PFU/mL)	97 (90–99)	99 (95–99)	78 (12–99)	98 (82–99)	98 (96–99)	90 (59–96)
RacL11 (5 × 10^6^ PFU/mL)	98 (94–99)	98 (98–99)	98 (97–99)	98 (97–99)	99 (98–99)	99 (96–99)
Thrombin (0.15 U/mL)	97 (91–99)	98 (92–99)	87 (3–95)	96 (94–98)	98 (97–99)	86 (6–98)

*The PRP was exposed to phosphate-buffered saline (PBS, negative control), thrombin (positive control), and low and high concentrations of bovine factor Xa and the Ab4 and RacL11 strains of EHV-1 as triggers for thrombin generation. Platelet activation was measured by P-selectin expression with flow cytometry. There were no significant differences in median P-selectin expression within treatments over time*.

**Table 3:** Median (range) percentage of platelets expressing P-selectin in platelet-rich plasma (PRP) from five horses before (T0) and 2 (T2) and 24 (T24) h after intravenous (IV) administration of placebo or 0.15 mg/kg apixaban.

**Agonist**	**P-selectin-positive platelets (%)**					
	**Placebo IV**			**Apixaban IV**		
	**T0**	**T2**	**T24**	**T0**	**T2**	**T24**
PBS	0 (0–1)	0 (0–1)	0 (0–0)	0 (0–0)	0 (0–1)	0 (0–0)
Factor Xa (0.1 ng/mL)	6 (0–99)	53 (0–98)	1 (0–96)	98 (4–99)	8 (0–99)	9 (1–96)
Factor Xa (1 ng/mL)	99 (98–99)	99 (98–99)	97 (75–99)	98 (98–99)	99 (98–99)	98 (86–99)
Ab4 (1 × 10^6^ PFU/mL)	8 (1–45)	13 (1–92)	1 (0–17)	6 (1–91)	2 (1–27)	1 (0–80)
Ab4 (5 × 10^6^ PFU/mL)	97 (45–98)	57 (6–99)	5 (2–95)	92 (1–98)	33 (3–91)	2 (1–83)
RacL11 (1 × 10^6^ PFU/mL)	99 (28–99)	99 (24–99)	98 (1–99)	87 (55–99)	86 (14–98)	96 (91–97)
RacL11 (5 × 10^6^ PFU/mL)	98 (95–99)	98 (98–99)	98 (96–99)	98 (96–99)	98 (93–98)	97 (93–98)
Thrombin (0.15 U/mL)	90 (49–97)	94 (35–98)	91 (31–97)	63 (36–98)	90 (60–95)	83 (36–87)

*The PRP was exposed to phosphate-buffered saline (PBS, negative control), thrombin (positive control), and low and high concentrations of bovine factor Xa and the Ab4 and RacL11 strains of EHV-1 as triggers for thrombin generation. Platelet activation was measured by P-selectin expression with flow cytometry. There were no significant differences in median P-selectin expression within treatments over time*.

### Measurement of Apixaban Concentrations With an Apixaban-Calibrated Anti-Xa Activity Assay

**Figure 7:**
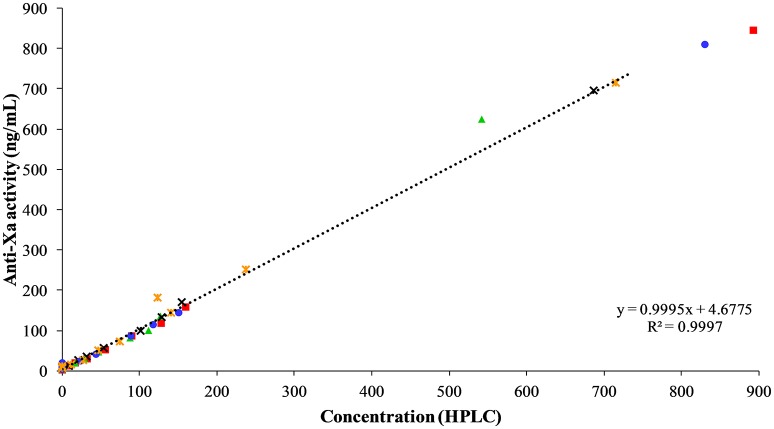
Correlation between apixaban-calibrated anti-Xa activity and apixaban concentration by UPLC-MS, both measured in ng/mL, at 0, 3, 30, and 45 min, and 1, 2, 3, 4, 6, 8, and 24 h after intravenous administration of apixaban at 0.15 mg/kg to five horses. Each horse has the same unique color and symbol at all time points for both treatments (horse 1: blue, •; horse 2: red, ■; horse 3: green, ▴; horse 4: orange, 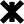; horse 5: black, ×).

The apixaban concentrations measured with the anti-Xa activity assay showed excellent correlation to the UPLC-MS method (*r*^2^ = 0.9997, *P* = < 0.0001, Figure 7). No change in anti-Xa activity was seen with the oral or IV placebo or oral dosing of apixaban. The median anti-Xa activity differed significantly from baseline only for the first hour after IV administration of apixaban (Table 4).

**Table 4:** Median (range) of apixaban-calibrated anti-Xa activity in citrate-anticoagulated platelet-poor plasma of five horses administered placebo or apixaban orally (PO, 0.2 mg/kg) or intravenously (IV, 0.15 mg/kg) before treatment, then at 3 (IV only), 15 (PO only), 30, and 45 min, and 1, 2, 3, 4, 6, 8, and 24 h after administration.

**Treatment**	**Anti-Xa activity (ng/mL)**										
	**0 min**	**3/15 min**	**30 min**	**45 min**	**1 h**	**2 h**	**3 h**	**4 h**	**6 h**	**8 h**	**24 h**
Placebo PO	6.5 (2.8–11.2)	5.2 (3.7–11.9)	6.6 (6.0–14)	6 (4.5–8.8)	6.6 (3.1–14.6)	7.3 (0.3–11.5)	5.4 (3.1–11.2)	7.2 (5.5–8.3)	5.8 (1.2–10.5)	6.2 (4–10.5)	6.5 (4.3–10.6)
Apixaban PO	8.7 (5.4–9.9)	7.4 (5.9–9.4)	7.5 (6.8–12.5)	9.9 (8.4–10.1)	10.9 (8.4–17)	9.7 (7.4–14.8)	8.7 (5.4–11.8)	9.3 (3.8–12)	10.7 (6.3–13.8)	8.7 (7.3–10.9)	7.6 (0–8.3)
Placebo IV	5.1 (2.4–9.8)	4.7 (3.6–8.6)	6.4 (3.9–9.5)	6.2 (3.7–8.4)	6.1 (4.5–9.3)	5.8 (2.1–7.8)	5.4 (3.1–6.9)	6.1 (5.6–21)	8.4 (4.8–9.7)	5.9 (4.6–8.5)	7.8 (2–8.7)
Apixaban IV	6.5 (2.1–7.4)	715^*^ (624–845)	158^*^ (135–251)	118^*^ (100–182)	86.5^*^ (81.8–144)	52.2 (41.1–72.7)	29.8 (24.4–50.7)	19.6 (19.3–26.6)	11.8 (8.8–15.4)	8.9 (7.9–11.6)	7.4 (4–8.2)

* *P < 0.05 in comparison with baseline (0 min)*.

### Clinical Pathologic Testing

There were no clinically apparent adverse effects associated with the administration of apixaban with oral and IV formulations in any horse. There were no significant changes in any hematologic test result 24 h after PO or IV administration of placebo or apixaban (Table 5). Some individual horses had test results that were slightly above or below the established reference intervals, but this likely represented normal biological variability. A low platelet count was seen in 1 horse at two different timepoints, but platelet clumps were noted in the blood smears. Similarly, there were no significant changes in any of the chosen biochemical test results 24 h after PO or IV administration of placebo or apixaban (Table 6). Individual horses showed variability in biochemical test results, with a few horses having transiently increased liver enzyme activities or decreased albumin concentrations at one or both timepoints. Again, we attributed this to normal biological variation.

**Table 5:** Mean ± standard deviation (range) of hematologic test results of five horses administered placebo or apixaban orally (PO, 0.2 mg/kg) or intravenously (IV, 0.15 mg/kg) before treatment (T0) and 24 h (T24) after placebo or drug administration.

**Test (SI unit)**	**Placebo PO**		**Apixaban PO**		**Placebo IV**		**Apixaban IV**		**Reference interval**
	**T0**	**T24**	**T0**	**T24**	**T0**	**T24**	**T0**	**T24**
RBC (× 10^12^/L)	7.1 ± 0.6 (6.4–7.8)	7.2 ± 0.5 (6.6–7.8)	7.1 ± 0.3 (6.7–7.6)	7.8 ± 0.4 (7.2–8.3)	7.3 ± 0.7 (6.3–8.2)	7.8 ± 0.4 (7.3–8.2)	7.4 ± 0.8 (6.5–8.4)	7.7 ± 0.7 (6.7–8.4)	6.6–9.7
HCT (L/L)	0.36 ± 0.03 (0.33–0.40)	0.38 ± 0.03 (0.35–0.42)	0.37 ± 0.03 (0.34–0.41)	0.40 ± 0.02 (0.38–0.42)	0.38 ± 0.02 (0.36–0.41)	0.41 ± 0.04 (0.37–0.47)	0.39 ± 0.03 (0.34–0.42)	0.40 ± 0.03 (0.37–0.46)	0.34–0.46
HGB (g/L)	128 ± 10 (116–139)	130 ± 11 (120–146)	130 ± 11 (120–147)	142 ± 9 (129–149)	135 ± 8 (127–145)	143 ± 14 (127–165)	135 ± 10 (122–146)	142 ± 12 (131–162)	118–159
WBC (× 10^9^/L)	6.4 ± 1.3 (4.8–8.4)	6.8 ± 1.2 (5.5–8.8)	6.1 ± 0.9 (5.1–7.2)	6.7 ± 0.9 (5.9–7.9)	6.6 ± 1.3 (5.4–8.6)	7.3 ± 0.8 (6.0–7.9)	6.8 ± 2.2 (5.1–10.5)	7.0 ± 1.1 (5.5–8.5)	5.2–10.1
NEUT (× 10^9^/L)	3.7 ± 0.7 (2.6–4.3)	3.9 ± 0.5 (3.0–4.3)	3.5 ± 0.5 (2.9–4.1)	3.9 ± 0.6 (3.1–4.6)	3.9 ± 0.3 (3.6–4.2)	4.2 ± 0.5 (3.7–5.0)	4.1 ± 1.3 (2.7–6.3)	4.1 ± 0.6 (3.2–4.7)	2.7–6.6
LYMPH (× 10^9^/L)	1.9 ± 0.7 (1.5–3.2)	2.2 ± 0.8 (1.4–3.5)	1.9 ± 0.5 (1.2–2.6)	2.0 ± 0.5 (1.3–2.7)	2.0 ± 1.0 (1.2–3.6)	2.2 ± 0.7 (1.5–3.3)	1.9 ± 0.9 (1.1–3.4)	2.0 ± 0.6 (1.6–3.1)	1.2–4.9
PLAT (× 10^9^/L)	116 ± 18 (89–132)	122 ± 13 (104–135)	118 ± 16 (105–141)	115 ± 21 (83–140)	128 ± 19 (99–146)	117 ± 9 (108–130)	124 ± 23 (104–163)	123 ± 15 (105–145)	94–232

*There was no statistical difference (P > 0.05) in any mean result comparing T0 to T24*.

*RBC, red blood cell count; HCT, hematocrit; HGB, hemoglobin; WBC, white blood cell count; NEUT, absolute automated neutrophil count; LYMPH, absolute automated lymphocyte count; PLAT, platelet count*.

**Table 6:** Median (range) of select biochemical test results of five horses administered placebo or 0.15–0.2 mg/kg apixaban orally (PO) or intravenously (IV) measured before (T0) and 24 h (T24) after the administration of placebo or drug.

**Test (SI unit)**	**Placebo PO**		**Apixaban PO**		**Placebo IV**		**Apixaban IV**		**Reference interval**
	**T0**	**T24**	**T0**	**T24**	**T0**	**T24**	**T0**	**T24**	
UN (mmol/L)	5.7 (5.7–7.8)	5.0 (4.6–6.8)	6.4 (5.4–7.1)	5.7 (4.6–6.4)	6.4 (5.4–8.6)	5.4 (4.3–6.8)	6.8 (6.1–7.8)	5.7 (5.4–7.5)	3.9–10.0
CREAT (μmol/L)	97 (88–106)	106 (97–115)	97 (88–106)	106 (97–115)	106 (97–115)	115 (106–115)	106 (97–106)	115 (106–115)	53–141
AST (U/L)	274 (269–316)	279 (259–305)	282 (263–289)	291 (266–309)	302 (259–312)	302 (264–304)	275 (226–301)	285 (235–310)	199–374
SDH (U/L)	8 (4–20)	4 (3–6)	5 (4–10)	4 (3–5)	3 (3–5)	3 (2–3)	4 (3–4)	3 (2–4)	0–11
GLDH (U/L)	6 (4–17)	5 (4–8)	6 (4–10)	4 (4–7)	5 (3–6)	4 (3–5)	4 (3–4)	3 (3–5)	1–8
GGT (U/L)	13 (10–15)	12 (10–14)	12 (7–13)	12 (9–13)	12 (7–14)	12 (9–12)	11 (10–13)	11 (10–13)	8–29
TP (g/L)	66 (65–71)	65 (64–69)	67 (62–70)	65 (63–72)	67 (64–74)	68 (64–73)	68 (66–71)	68 (67–72)	57–77
ALB (g/L)	31 (30–34)	31 (30–34)	32 (29–32)	32 (29–33)	32 (30–35)	32 (31–34)	32 (30–34)	32 (30–35)	30–37
GLOB (g/L)	36 (31–38)	35 (30–36)	35 (30–38)	36 (30–39)	35 (31–39)	37 (31–39)	36 (32–38)	37 (32–40)	24–44
SAA^*^ (mg/L)	5 (5–7)	5 (5–5)	5 (5–7)	5 (5–5)	5 (5–6)	5 (5–8)	5 (5–9)	8 (5–12)	5–20

*There was no statistical difference (P > 0.05) in any mean result comparing T0 to T24. UN, urea nitrogen; CREAT, creatinine; AST, aspartate aminotransferase; SDH, sorbitol dehydrogenase; GLDH, glutamate dehydrogenase; GGT, gamma glutamyl transferase; TP, total protein; ALB, albumin; GLOB, globulins; SAA, serum amyloid A*.

* *5 mg/L is the lower limit of detection for this assay*.

## Discussion

The results of our study show that a single oral dose of apixaban at 0.2 mg/kg was not absorbed by horses. The chosen dose was previously studied in cats and showed a high bioavailability of 85% (25), which is comparable to that in dogs (26). A lower availability of 50% is evident after oral administration in rats, chimpanzees, and humans (26, 37). In contrast, rabbits show an oral absorption of only 3%, even with higher doses in comparison with the other species studied this far (10 mg/kg vs. doses varying between 0.2 and 5 mg/kg) (27). The lack of oral absorption in horses could be attributed to different factors. The dose used in this study may have been insufficient to generate detectable plasma levels. Apixaban is a substrate for P-glycoprotein (38), which is highly expressed in the small intestine of horses (39). This transmembrane protein inhibits absorption of several other drugs (40) and may be preventing apixaban absorption in horses. Horses may also have a high first-passage metabolism which could greatly reduce bioavailability, similar to that described for rabbits (41).

We found that apixaban had a small volume of distribution (~90 mL/kg) after IV administration. This finding likely reflects the plasma volume in a healthy horse, which is consistent for a drug with high protein binding and confined to the plasma. A small volume of distribution is a desirable characteristic in a drug that should remain in the central compartment, such as anticoagulants (37). High protein binding was also seen in other species, with the exception of rabbits (37), in which ~60% of the apixaban was protein bound. The 152 mL/h/kg rate of clearance of apixaban is similar to the glomerular filtration rate in horses (42), suggesting that the urinary route is the major elimination pathway in this species. In contrast, the fecal route of elimination accounts for more than 54 and 46% of the dose in other animals and humans, respectively (41). The elimination half-life of 1.3 h in the horses of this study is similar to that reported for cats, with a 1.1 h half-life after a 0.2 mg/kg IV dose (25), and longer than that observed in rabbits, which is 0.6 h after a 2.5 mg/kg IV dose (37).

The anti-Xa assay used in this study is an excellent surrogate for the “gold standard” measurement of apixaban concentrations by UPLC-MS. For pharmacodynamic analysis, we chose to use the dPT vs. the activated partial thromboplastin time (APTT) because preliminary studies in the Comparative Coagulation Laboratory showed higher variability and lack of discernible prolongation with the APTT in this cohort (data not shown). However, our results showed that, with the PT reagent used in this study, the dPT could not be used for pharmacodynamic analysis due to the lack of a discernible relationship between the dPT and UPLC-MS concentrations. This was attributed to the wide inter- and intraindividual variability in clotting times in the horses of this study, which has also been reported by others (43). The dPT was only weakly correlated to drug concentrations in cats given 3 different single oral doses of rivaroxaban (*r* = 0.53, *P* < 0.001). The latter study found that there was no correlation between rivoraxaban concentrations and the APTT (29). In humans, the sensitivity of standard screening coagulation assays to the inhibitory effect of apixaban varies between studies and is reagent dependent. Kanemoto, Kuhara (43) showed that PT results were significantly prolonged in a concentration-dependent manner and were linear in the apixaban concentration range of 0–500 ng/mL in spiked pooled human plasma. A separate study of apixaban and rivaroxaban (0–200 ng/mL) in spiked human plasma showed that none of the seven tested APTT reagents and only one of six tested PT reagents were sensitive to apixaban (44). Douxfils, Chatelain (45) compared the PT, dPT, and APTT in spiked human plasma with apixaban concentrations ranging from 5 to 500 ng/mL. Although all three tests showed a concentration-dependent prolongation of coagulation times, none of these tests could predict apixaban pharmacodynamic properties, as we found in our study with the dPT. Our results indicate that monitoring of apixaban should be done with the anti-Xa activity assay vs. standard or modified coagulation screening assays.

Our study also showed that apixaban, despite achieving measurable concentrations *in vivo* after IV administration, had no effect on *ex vivo* EHV-1-induced platelet activation. Individual horses showed substantial variability in *ex vivo* induced platelet activation, which we have previously seen in the same horses when they were given heparin-based anticoagulants as part of a different clinical trial (36). However, the latter study showed consistent inhibition of *ex vivo* EHV-1-induced platelet activation, unlike this study herein, indicating that apixaban, even if given IV, will likely not be an effective anticoagulant for blocking thrombin generation induced by EHV-1 or factor Xa *in vivo*. It is possible that if we had tested horses at the time of peak drug concentrations or earlier than the 2 h chosen timepoint, that we would have detected an inhibitory effect of apixaban on *ex vivo* EHV-1-induced platelet activation.

In conclusion, our study showed that apixaban, an anticoagulant that specifically inhibits activated factor X, was not orally absorbed by horses at a dose of 0.2 mg/kg and therefore cannot be used at this dose as an oral anticoagulant in horses. A comparable IV dose of 0.15 mg/kg, even though it achieved relatively high plasma concentrations for up to 2 h after administration, was ineffective at inhibiting thrombin generation induced by EHV-1, using platelet activation as readout. It is possible that higher doses of apixaban given orally will be absorbed, however without additional data on efficacy, we believe it unlikely that this drug would be affordable or effective oral anticoagulant in horses, leaving us with the heparin-based anticoagulants as our current best alternative for inhibiting thrombin generation *ex vivo*.

## Ethics Statement

The study was approved by the Institutional Laboratory Animal Care and Use Committee at Cornell University (protocol No. 2017-0030), accordingly to AAALAC (Association for Assessment and Accreditation of Laboratory Animal Care) guidelines.

## Author Contributions

TS conceived and designed the study, collected blood samples, did the flow cytometric assays, reviewed and interpreted the data, and co-wrote the manuscript. PS helped with study design and organization, prepared the virus and drugs, performed some of the flow cytometric assays, reviewed and interpreted the data, and wrote the manuscript. MB helped with study design, data interpretation, and oversaw the anti-Xa activity assays. TD and SN helped design the study, administered the drugs and collected blood samples. IB performed the HPLC analysis for apixaban. MP performed the pharmacokinetics analysis and helped with study design. All authors read and edited the manuscript.

### Conflict of Interest Statement

The authors declare that the research was conducted in the absence of any commercial or financial relationships that could be construed as a potential conflict of interest.

## Abbreviations

PBS: phosphate-buffered saline
DOAC: direct oral anticoagulant
DMSO: dimethyl sulfoxide
EHV: equid herpesvirus
PPP: platelet-poor plasma
PRP: platelet-rich plasma
PFU: plaque forming units
Xa: activated factor X
